# Evaluation and Comparison of Choroidal Thickness in Patients with Behçet Disease with versus without Ocular Involvement

**DOI:** 10.18502/jovr.v16i2.9083

**Published:** 2021-04-29

**Authors:** Seyedeh Maryam Hosseini, Negar Morrovatdar, Armin Hemati, Mozghan Dolatkhah, Elham Bakhtiari, Zahra Mirfeizi, Mahdieh Azimizadeh

**Affiliations:** Department of Ophthalmology, Mashhad University of Medical Sciences, Mashhad, Iran

**Keywords:** Behçet's Disease, Behçet's Uveitis, Choroidal Thickness, Enhanced Depth Imaging Optical Coherence Tomography (EDI-OCT), Ocular Behçet, Wide Field Fluorescein Angiography

## Abstract

**Purpose:**

To assess the subfoveal choroidal thickness (SFCT) in patients with Behçet disease (BD) and compare the SFCT in patients with and without ocular BD (OBD) and between patients with active and quiescent phases of the Behçet's posterior uveitis.

**Methods:**

This prospective cross-sectional study was conducted on patients with BD (*n* = 51) between October 2016 and October 2018. Complete ocular examinations including slit lamp biomicroscopy and fundus examination with dilated pupils were performed for all patients. The SFCT values were compared between patients with and without OBD. Enhanced depth imaging optical coherence tomography (EDI–OCT) was done to measure the SFCT, and wide field fundus fluorescein angiography (WF-FAG) was performed to evaluate the ocular involvement and determine the active or quiescent phases of the Behçet's posterior uveitis. The correlation between the changes of SFCT and the WF-FAG scores was assessed.

**Results:**

One hundred and two eyes of 51 patients with BD, aged 29 to 52 years were studied. Of these, 23 patients were male. The mean age ± standard deviation in patients with OBD and patients without ocular involvement was 38.71 ± 7.8 and 36.22 ± 10.59 years (*P* = 0.259) respectively. The mean SFCT in patients with OBD was significantly greater than in patients without OBD (364.17 ± 93.34 vs 320.43 ± 56.70 µm; *P* = 0.008). The difference of mean SFCT between the active compared to quiescent phase was not statistically significant when only WF-FAG criteria were considered for activity (368.12 ± 104.591 vs 354.57 ± 58.701 µm, *P* = 0.579). However, when the disease activity was considered based on both WF-FAG and ocular exam findings, SFCT in the active group was higher than the inactive group (393.04 ± 94.88 vs 351.65 ± 58.63 µm, *P* = 0.060). This difference did not reach statistical significance, but it was clinically relevant.

**Conclusion:**

Choroidal thickness was significantly increased in BD patients with ocular involvement; therefore, EDI-OCT could be a noninvasive test for evaluation of ocular involvement in patients with BD. The increased SFCT was not an indicative of activity in OBD; however, it could predict possible ocular involvement throughout the disease course.

Seyedeh Maryam Hosseini, MD; Negar Morrovatdar, MD; Armin Hemati, MD; Mozghan Dolatkhah, MD Elham Bakhtiari, MD; Zahra Mirfeizi, MD; Mahdieh Azimizadeh, MD

Choroidal Thickness in Patients with Behçet Disease

##  INTRODUCTION 

Behçet disease (BD) is a chronic, recurrent, inflammatory, multisystem disease characterized by occlusive and necrotizing vasculitis.^[1,2,3]^ The prevalence of BD is estimated to be 10.3 per 100,000 individuals and is higher in Middle East, Far East, and Mediterranean countries.^[4,5]^ Different classification criteria exist for BD, including the new “international criteria for BD.” Ocular involvement is a common consequence of BD as 70–90% of the patients experience ocular impairments that are presented in different forms including posterior uveitis, retinal vasculitis, anterior uveitis, and optic neuropathy.^[6]^ Loss of vision has been correlated with the disease duration.^[5]^ In 50–93% of the patients with ocular involvement, the posterior segment eye diseases are observed.^[7]^ Choroidal vessels can be involved in addition to retinal vessels as the primary focus or secondary to retinal inflammation.^[8]^ Recurrent inflammatory phases can severely impair the retinal structure and function and result in visual loss.^[9,10,11,12,13]^ Early detection of posterior segment involvement during the early phase of the disease is necessary for development of an effective treatment strategy.^[9]^ Fundus fluorescein angiography (FFA) is the standard method for monitoring and evaluating the progression of ocular Behçet disease (OBD).^[10,11,13]^ This imaging modality has shown high sensitivity for detection of retinal vasculitis in BD cases.^[10]^ Indocyanine green angiography (ICGA) has been proposed as an alternative modality which evaluates choroidal circulation; however, it does not disclose the retinal circulation or evidence of retinal vasculitis as clear as FFA does.^[14,15,16]^ Several studies have investigated the choroidal thickness analysis with EDI-OCT in patients with OBD.^[6,8,14,17,18]^ Their findings are controversial regarding the effects of OBD disease on choroidal thickness. While some studies have reported that choroid thickness increases in OBD patients,^[6,8,17,18]^ others have demonstrated that OBD decreases the choroidal thickness.^[14]^ These discrepancies in choroidal thickness could be attributed to the non-homogeneity in patients' features in terms of activity, anatomical involvement, and OBD duration.^[8]^

In this study, we tried to investigate the subfoveal choroidal thickness (SFCT) in Behçet's patients with and without ocular involvement using EDI-OCT modality. We also compared SFCT between active and quiescent phases of OBD and evaluated the correlations between the changes of SFCT in EDI-OCT and the WF-FAG scores.

##  METHODS

This prospective cross-sectional study was conducted on patients with BD (*n* = 51; eyes: 102) who had been referred to the uveitis clinic of Khatam-Al-Anbia tertiary eye center, affiliated to Mashhad University of Medical Sciences (MUMS) between October 2016 and October 2018. The study was approved by the local institutional review board and ethics committee of MUMS, Mashhad, Iran, and was in accordance with the ethical standards and regulations of the Declaration of Helsinki.

The inclusion criteria of the study were patients older than 18 years with definite diagnosis of BD according to the diagnostic criteria of the International Study Group for BD with or without ocular involvement. Patients who met the inclusion criteria were evaluated in three groups:

(i) Patients with ocular involvement and active uveitis

(ii) Patients with ocular involvement who were in remission

(iii) Patients without ocular involvement in examination or WF-FAG

Behçet's patients with posterior, intermediate, or pan-uveitis were included in the study. Evidence of posterior uveitis was documented either on examination by the presence of inflammatory cells in the vitreous and/or retinitis, or retinal vasculitis manifesting as perivascular sheathing, or vascular leakage and/or occlusion on fluorescein angiograms.

The exclusion criteria were age over 55 years, history of intraocular surgery, chronic systemic diseases such as diabetes mellitus, hypertension, vascular diseases and other rheumatologic involvement, any ocular diseases like age-related macular degeneration (AMD), central serous choroidopathy (CSC), high refractive error (spherical equivalent >±3.00D), any media opacity precluding image acquisition, allergy to fluorescein and non-compliance with follow-up. Patients with only anterior uveitis were excluded.

Demographic information of the patients and the ophthalmologic examinations data including best-corrected visual acuity (BCVA), slit lamp biomicroscopy, tonometry, and dilated indirect ophthalmoscopy were collected for further analysis. BCVA was measured by decimal Snellen scale and then converted to the logarithm of the minimal angle of resolution (log MAR) for statistical analysis. All patients underwent EDI-OCT and WF-FAG imaging modalities.

The SFCT was measured using digital caliper provided by EDI-OCT (Heidelberg Engineering, Heidelberg, Germany). Choroidal thickness was measured from the outer portion of the hyper-reflective line corresponding to the retina pigment epithelium (RPE) to the inner surface of the sclera. To avoid diurnal variations, measurements of SFCT with EDI-OCT were performed from 9 am to 1 pm. To reduce the bias, two expert ophthalmologists who were masked to the patient grouping measured the choroidal thickness independently in each eye. Any case with 15% difference in the two measurements by the two experts was excluded from the study. All patients underwent WF-FAG (Heidelberg Engineering, Heidelberg, Germany) procedure following intravenous (IV) administration of 2.5 mL sodium fluorescein 10% (Fluorescite; Alcon, Inc, Fort Worth, TX). The angiographic scoring method described by Tugal-Tutkun et al^[19]^ was used to score the FA. In this scoring method, they assigned scores to the fluorescein and ICG angiographic signs that represented active inflammatory process in the posterior segment. A total maximum score of 40 was assigned to the FA signs, including optic disc hyperfluorescence, macular edema, retinal vascular staining and/or leakage, capillary leakage, retinal capillary nonperfusion, neovascularization of the optic disc, neovascularization elsewhere, pinpoint leaks, and retinal staining and/or subretinal pooling. A total maximum score of 20 was assigned to the ICGA signs, including early stromal vessel hyperfluorescence, choroidal vasculitis, dark dots or areas (excluding atrophy), and optic disc hyperfluorescence.

##  RESULTS

Table 1 shows demographic information, age, gender, BCVA, and SFCT in the studied patients. There was no significant difference in terms of gender and age between the groups with and without ocular involvement.

**Table 1:** Demographic and clinical finding of patients


	**Without ocular involvement**	**With ocular involvement**	**** ***P*** **-value**
**Age**	38.71 ± 7.8 (29–52 years)	36.22 ± 10.59 (21–55 years)	0.259
**Sex (M:F)**	5:9	16:19	0.241
**BCVA**	0.1403 ± 0.24	0.0035 ± 0.018	0.005
**Mean SFCT (µm)**	320.43 ± 567	364.17 ± 93.34	0.008
BCVA, best-corrected visual acuity (logMAR); SFCT, sub-foveal choroidal thickness			

**Table 2:** Demographic and clinical finding of patients with ocular involvement only in WF-FAG


	**Active uveitis**	**Inactive uveitis**	**** ***P*** **-value**
**AGE**	35.60 ± 10.906	37.76 ± 9.833	0.433
**Sex (M:F)**	33:20	3:18	0.001
**BCVA**	0.2002 ± 0.28	0.0120 ± 0.038	0.003
**Duration of disease (yr)**	5.018 ± 6.28	5.119 ± 3.65	0.945
**5Mean SFCT (µm)**	368.12 ± 104.591	354.57 ± 58.701	0.579
WF-FAG, wide field fundus fluorescein angiography; BCVA, best corrected visual acuity; SFCT, subfoveal choroidal thickness			

**Table 3:** Demographic and clinical finding of patients with ocular involvement in both WF-FAG and clinical exam


	**Active uveitis**	**Inactive uveitis**	**** ***P*** **-value**
**Number (eyes)**	26	20	
**AGE**	36.56 ± 8.85	36.34 ± 9.573	0.385
**SEX (M:F)**	12:14	8:12	0.259
**BCVA**	0.2210 ± 0.14	0.0095 ± 0.056	0.03
**Mean SFCT (µm)**	393.04 ± 94.88	351.65 ± 58.63	0.060
BCVA, best corrected visual acuity; SFCT, subfoveal choroidal thickness			

Fifty-one patients (102 eyes) participated in this study (23 males and 28 females). Twenty-eight eyes showed no ocular involvement. Seventy-four eyes had ocular involvement in WF-FAG or clinical examination; of these, twenty-eight eyes (37.8% ) demonstrated active ocular inflammation manifesting as posterior uveitis in ophthalmic examination, whereas the uveitis was quiescent in 46 eyes corresponding with 62.2% of patients with ocular involvement. However, in the WF-FAG evaluation, 53 eyes (71.6% of patients with OBD) had active uveitis (score >0) and 21 eyes (28.4%) showed inactive uveitis (score of 0) (WF-FAG score range, 1–29). In evaluation of both ophthalmic examination and WF-FAG in patients with ocular involvement, 26 eyes (36.5%) showed to be active and 20 eyes (27%) were inactive.

The mean age of patients in the group without ocular involvement was 38.71 ± 7.8 years (29–52 years) and in the group with ocular involvement was 36.22 ± 10.59 (21–55 years) (*P* = 0.259). The mean duration of BD was 5.34 ± 4.6 years in all patients. The minimum duration of disease was in new cases and the maximum duration was 22 years.

The mean BCVA (Log MAR) in the BD patients with and without ocular involvement was 0.1403 ± 0.24 and 0.0035 ± 0.018 (*P* = 0.005), respectively.

There was no eye with >15% variation in measurement of SFCT between the ophthalmologists. The mean SFCT in patients without ocular involvement measured on EDI-OCT was 320.43 ± 56.70 µm (222–452 µm) and in patients with ocular involvement on clinical examination or WF-FAG was 364 ± 93.34 µm (88–519µm) (*P* = 0.008). The Mann–Whitney test showed significant correlations (*P* = 0.008) between SFCT and ocular involvement (Table 1).

The SFCT measured by EDI-OCT in the patients with active OBD demonstrating only in WF-FAG was 368.12 ± 16.64 µm (range: 88–519 µm), and in inactive eyes it was 354.57 ± 58.701 (range, 222–452 µm) (*P* = 0.579) (Table 2). The SFCT values did not show significant correlation with WF-FAG activity (*P* = 0.579). SFCT values in the active group according to both WF-FAG and clinical examination was higher than in the inactive group but it was not statistically significant regardless of the clinical relevance (393.04 ± 94.88 vs 351.65 ± 58.63) *P* = 0.060 (Table 3).

Spearman correlation test showed significant negative correlation between SFCT and BD duration (P = 0.013, r = –0.249) indicating inverse relationship between BD duration and SFCT.

The mean ± SD WF-FAG score of active uveitis cases was 18 ± 7. There was an association between total WF-FAG score and mean SFCT in EDI-OCT so that higher total WF-FAG scores resulted in higher mean SFCT values, but the association was not statistically significant (*P* = 0.579).

In addition, we evaluated the association between BCVA and gender in the patients. The mean Log MAR BCVA was 0.153 and 0.057 in males and females, respectively. BCVA was significantly better in females and the Mann–Whitney test showed significant correlations between BCVA and gender (*P* = 0.002).

##  DISCUSSION

In the current study, patients with OBD had thicker choroid compared to patients without ocular involvement, and the subfoveal choroid was thicker in patients with active disease compared to those with quiescent disease. To the best of our knowledge, this is the first report of choroidal thickness measurement in BD with and without ocular involvement in Iran.

Choroidal involvement in OBD has been demonstrated in histopathology, ophthalmic ultrasonography, ICGA, and WF-FAG.^[20,21,22]^

EDI–OCT is a technique that provides noninvasive, *in vivo*, cross-sectional, histologic information of the choroid and retina with high resolution.^[12,16]^

WF-FAG is the gold standard technique for evaluation of posterior involvement in OBD. However, this technique is poorly sensitive for detailed evaluation of the choroidal circulation.^[14]^ ICGA has been proposed for the assessment of choroidal circulation, but it shows low sensitivity for the evaluation of retinal circulation.^[15,16]^

We investigated mean SFCT with EDI–OCT in 102 eyes of 51 patients with definite BD and found that choroid was thicker in patients with ocular involvement in both active and inactive uveitic phases compared to the choroidal thickness in patients without ocular involvement. SFCT was also greater during an active uveitis in comparison to the cases in remission, but the difference was not statistically significant. This can be explained by either the possible subclinical involvement of the choroid in quiescent phase that results in an increase in the SFCT or that attacks of uveitis may cause lasting changes in the choroidal structure and thickness. The mean age of patients with and without ocular involvement did not significantly differ and the age of patients in this study was similar to the age of patients in previously conducted studies that were in their third and fourth decades of life.^[23]^

Previous studies evaluated choroidal changes by EDI–OCT in BD. Ishikawa et al^[17]^ and Kim et al^[6,9]^ reported that SFCT in the active phase of OBD was significantly greater than in remission phase. Moreover, the choroidal thickness in both active and remission phases of OBD was greater than the healthy subjects. This difference could be attributed to the subclinical inflammatory activity of the choroid during the quiescent phase, which could exacerbate, leading to an acute recurrent attack of uveitis.^[6]^ Their findings correlate with the findings of the current study. Results of the study by Atas et al also support our findings^[18]^. They reported that the SFCT in the eyes in the remission phase of OBD was greater than the healthy control eyes, but the difference was not statistically significant. In contrast, some studies contradicted our findings.^[8]^ Coskun et al^[14]^ demonstrated that the choroid was significantly thinner in the eyes of Behçet patients with active posterior uveitis compared to patients in remission and control healthy individuals after excluding patients with macular edema. They concluded that because of long duration of eye disease in the studied patients (4.1 years), prolonged inflammation resulted in choroidal fibrosis and shrinkage and decreased thickness.^[14]^

Onal et al analyzed the morphology of subfoveal choroid during an active OBD phase.^[8]^ They quantitatively segmented the choroid into choroidal stroma and choroidal vessel lumen in EDI–OCT images collected from patients with <4 year history of BD and acute posterior uveitis. Patients with OBD had a significantly higher choroidal stroma to choroidal vessel lumen compared to the healthy control group. Choroidal stromal expansion in eyes with active OBD was observed. However, choroidal stroma expansion was not associated with thickening of the choroid.^[8]^

Interestingly, Kim et al reported no significant difference in SFCT between the two eyes of BD patients with unilateral active uveitis because the choroid thickness was greater than the normal values in the uninvolved fellow eyes.^[6]^

The discrepancies in choroidal thickness in these studies could be attributed to the non-homogeneity in activity, anatomical involvement, and duration of OBD.^[8]^

We used the wide field angiographic scoring system to score WF-FAG^[19,24,25,26]^ and observed an association between choroidal thickness and WF-FAG activity score so that higher WF-FAG activity results in greater SFCT but the association was not statistically significant. The results of the current study contradicted the findings of Kim et al^[6]^ and Onal et al^[8]^ who reported significant correlations between SFCT values and total WF-FAG score, retinal vascular staining, and/or leakage.

The other interesting finding of the present study was the negative correlation between choroidal thickness and duration of the disease; the plausible explanation for this finding may be replacement of choroidal stroma by fibrosis in the chronic phase of BD that results in thinning of choroid in cases with longer duration of OBD in contrast to early involved patients.^[14]^ In the current study, BCVA was significantly better in females, which could be explained by the more severe course and more frequent involvement of the posterior segment in male patients with Behcet ocular involvement.^[5]^

In conclusion, our findings showed a significant change of SFCT in BD. Moreover, this study demonstrated that EDI–OCT modality is useful for evaluating the subclinical choroidal involvement even during the quiescent phase of the OBD. This technique can be used to monitor the activity of disease in association with the angiographic findings.^[6]^

This study has some limitations including small sample size and non-homogeneity of cases. Although EDI–OCT is a good modality for assessment of ocular involvement in BD patients, the commercially available tools can not automatically calculate the SFCT; manual measurements are tedious and time-consuming, and delineating the choroidal–scleral interface in subjects with a thick choroid is difficult. Moreover, opaque ocular media due to significant vitreous haziness and/or cataract impedes smooth acquisition of the images. Future developments in SS-OCT may provide more effective assessment of the choroid in ocular Behcet disease.

##  Financial Support and Sponsorship

None.

##  Conflicts of Interest

The authors have no conflicts of interest to declare.
